# Variable-resolution maps (VRMs) in *CCTBX* and *Phenix*: accounting for local resolution in cryoEM

**DOI:** 10.1107/S2052252526008201

**Published:** 2026-10-15

**Authors:** Pavel V. Afonine, Paul D. Adams, Alexandre G. Urzhumtsev

**Affiliations:** a Molecular Biophysics & Integrated Bioimaging Division Lawrence Berkeley National Laboratory Berkeley CA94720-8235 USA; b Department of Bioengineering University of California Berkeley Berkeley CA94720 USA; c Centre for Integrative Biology Department of Integrated Structural Biology IGBMC (Institute of Genetics and of Molecular and Cellular Biology) Centre National de la Recherche Scientifique (CNRS) UMR 7104/Institut National de la Santé de la Recherche Médicale (Inserm) U964/Université de Strasbourg Illkirch France; d Department of Physics Université de Lorraine Vandoeuvre-lès-Nancy 54506 France; University of British Columbia, Canada

**Keywords:** variable-resolution maps, atomic images, finite resolution, refinement, Fourier ripples, analytic approximations

## Abstract

Calculation of density maps from atomic models is essential for structural studies using crystallography and electron cryo-microscopy (cryoEM). These maps serve various purposes, including atomic model building, refinement, visualization and validation. However, accurately comparing model-calculated maps to experimental data poses challenges, particularly because the resolution of cryoEM experimental maps varies across the map. Traditional crystallography methods generate finite-resolution maps with uniform resolution throughout the unit cell volume, while most modern software in cryoEM employs Gaussian-like functions to generate these maps, which does not adequately account for atomic model parameters and resolution. Recent work by Urzhumtsev & Lunin [*IUCrJ* (2022), **9**, 728–734] introduces a novel method for computing atomic model maps that incorporate local resolution and can be expressed as analytically differentiable functions of atomic parameters. This approach enhances the accuracy of maps simulated from atomic models, allowing them to better reproduce features of the experimental maps. In this paper, we detail the implementation of this method in *CCTBX* and *Phenix*.

## Abstract 2

New tools implemented in *CCTBX* and *Phenix* allow the calculation of variable-resolution maps through a sum of finite-resolution atomic images expressed as analytic functions of all atomic parameters, along with their associated local resolution.

## 1. Introduction

Calculation of density maps from atomic models is fundamental to structural studies in crystallography and electron cryo-microscopy (cryoEM). These maps represent the distribution of electron density (X-ray crystallography), nuclear density (neutron crystallography), or electrostatic scattering potential (electron diffraction or cryoEM) for a given model. In structural biology, model-calculated maps are used for various goals and some recent examples are atomic model building (Mostosi *et al.*, 2020 ▸; Pfab *et al.*, 2021 ▸), flexible fitting (Trabuco *et al.*, 2008 ▸; Kirmizialtin *et al.*, 2015 ▸), refinement (DiMaio *et al.*, 2009 ▸; DiMaio *et al.*, 2015 ▸; Afonine *et al.*, 2018*b* ▸), map improvement (He *et al.*, 2023 ▸), ligand identification (Robertson *et al.*, 2020 ▸), validation (Afonine *et al.*, 2018*a* ▸; Pintilie *et al.*, 2020 ▸), structure prediction (Si *et al.*, 2020 ▸) and computer molecular graphics (Meng *et al.*, 2023 ▸). In this context, model-calculated maps are either matched against experimentally derived maps to assess, or enhance, the fit of the atomic model to observed data, or serve as training data for deep learning applications, or as temporary objects to speed up calculations (Sayre, 1951 ▸). Consequently, the ability to accurately and efficiently compute density maps from atomic models is important.

A proper comparison between a model and experimental data requires that both are expressed in compatible terms. Since experimental maps always have finite resolution, model-calculated maps must match this resolution. In crystallography, finite-resolution maps are usually computed by a three-step procedure. First, the exact density distribution is generated by summing individual atomic densities. Then corresponding Fourier coefficients are calculated and truncated at the experimental resolution limit. Finally, this truncated set of Fourier coefficients is converted back into a map that exhibits a uniform resolution aligned with that of the experimental data. This procedure is hardly applicable in cryoEM where the resolution of the experimental map usually varies over the map (*e.g.* Cardone *et al.*, 2013 ▸).

**Figure 1:**
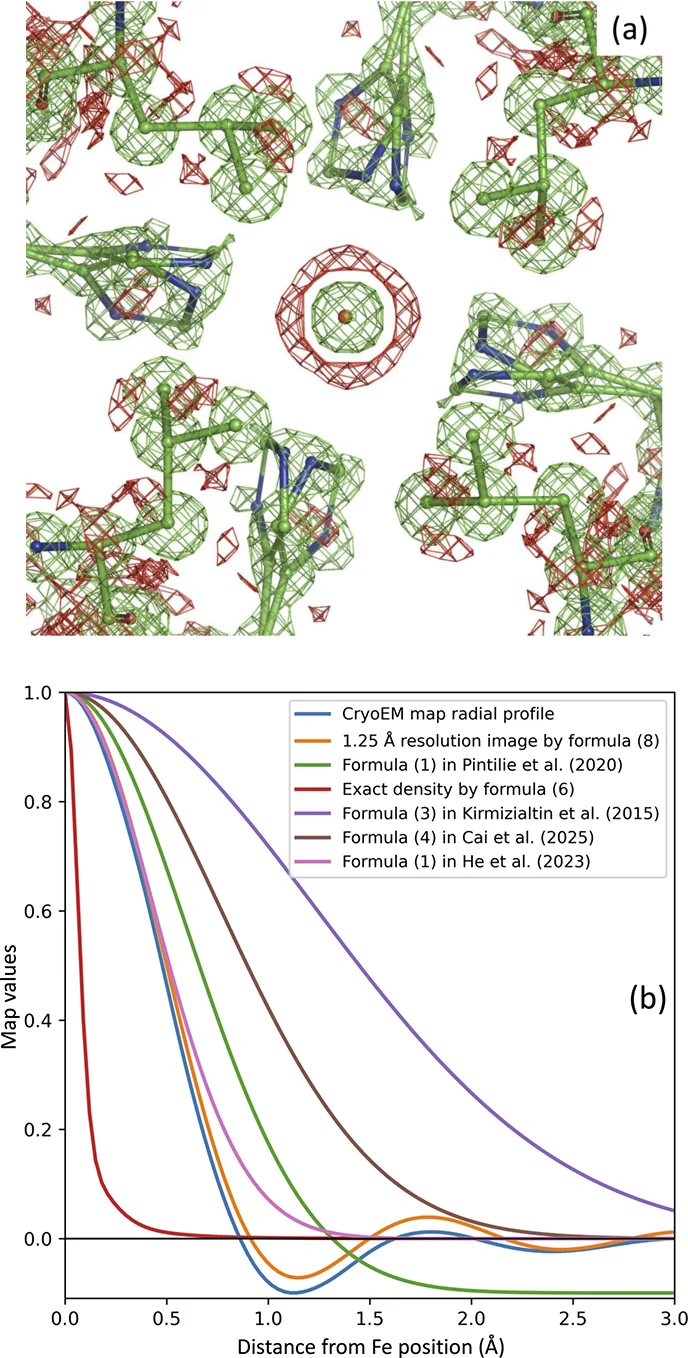
(*a*) CryoEM model of mouse heavy-chain apoferritin (PDB code: 8rqb), focusing on Fe 201 in chain A, and corresponding experimental map (EMDB code: 19436) contoured at ±1.5 units. (*b*) Radial components of the experimental cryoEM map around Fe (blue curve), exact density (red curve) and its finite-resolution image computed using (8) (orange curve), and of several maps calculated for an isolated Fe atom. All maps normalized by their values at the origin (maximum value). See the main text for details.

In cryoEM, current software typically generates atomic model maps by summing contributions from individual atoms within a specified surrounding volume. Most programs use Gaussian-like functions (Mooij *et al.*, 2006 ▸; Topf *et al.*, 2008 ▸; DiMaio *et al.*, 2009 ▸; DiMaio *et al.*, 2015 ▸; Kirmizialtin *et al.*, 2015 ▸; Pintilie *et al.*, 2020 ▸; Zhang *et al.*, 2020 ▸; Blau *et al.*, 2023 ▸; He *et al.*, 2023 ▸; Meng *et al.*, 2023 ▸; Cai *et al.*, 2025 ▸; Selvaraj *et al.*, 2025 ▸), following the work of Costain (1941 ▸) and Booth (1946 ▸), who noted that ‘*Examination of the shape of the atomic peaks derived from a number of Fourier syntheses shows that the radial density distribution can be closely represented by the [Gaussian] function.*’ Additionally, machine learning techniques can also be employed to generate model maps (Zhang *et al.*, 2025 ▸). These model map calculations present two issues. First, they (except partially DiMaio *et al.*, 2015 ▸) do not explicitly account for atomic model parameters such as occupancy, atomic displacement parameters (ADPs), chemical element types and charges. Second, these models assume that atomic shapes at finite resolution manifest solely as positive density peaks, aligning with Diamond’s (1971 ▸) assertion that ‘*Theoretically, therefore, the image of an isolated atom … will not be distinguishable from Gaussian to an extent worth characterizing*.’ In reality, finite-resolution atomic images oscillate with both positive and negative ripples. This becomes evident when the experimental map is displayed with both positive and negative contours, in contrast to the customary positive-only representation. The central peak corresponding to the Fe ion in the cryoEM map for mouse heavy-chain apoferritin (Küçükoğlu *et al.*, 2024 ▸), along with the subsequent dip, clearly illustrates this point [Fig. 1 ▸(*a*), compare blue and orange lines in Fig. 1 ▸(*b*); here the orange curve shows the radial component of the inverse three-dimensional Fourier transform of the Fe ion scattering function truncated at the resolution 1.25 Å]. Failing to account for these ripples in the model map can degrade molecular images [Fig. 1 ▸(*c*) in Urzhumtsev & Lunin, 2022 ▸] and result in incorrect values of the atomic displacement parameter obtained by real-space refinement of atomic models (Lunin & Lunina, 2025 ▸). Also, using a simplified model (Afonine *et al.*, 2018*b* ▸) requires strong geometric restraints bias when refining atomic positions. To model finite-resolution atomic images more accurately, Chapman (1995 ▸) and Chapman *et al.* (2013 ▸) employed atomic images derived from finite-resolution Fourier transforms of piecewise approximations to the respective atomic scattering functions, though this method appears computationally intensive.

We emphasize that atomic models and their parameters, such as atom types, coordinates, scattering properties, and disorder descriptors such as occupancies and displacement parameters, are intended to describe the physical and chemical properties of the molecules and their states in the samples under investigation. In addition, atomic model parameterization can, in principle, account for the limited resolution of the experimental data, which is the subject of this work. Limited resolution is one of many artifacts present in the data and is not a property of true structures in nature.

Recently, Urzhumtsev & Lunin (2022 ▸) introduced a method that allows for the computation of atomic model maps that account for local resolution and are expressed as analytically differentiable functions of atomic coordinates, isotropic atomic displacement parameter, occupancy and local resolution. This readily enables their use in real-space refinement and machine learning training. In this approach, the entire variable-resolution model map is computed as a sum of individual atomic contributions, each one representing the atom’s image at a specified resolution 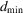 approximated by
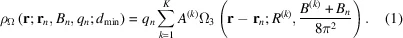
Here the function
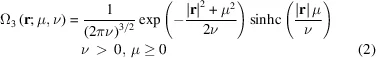
(Urzhumtsev & Lunin, 2024 ▸) can describe both the peak in the origin and Fourier ripples. In formula (1), 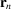 is the atomic center, 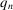 is the occupancy and 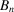 is the isotropic displacement parameter of the atom *n*. For a given resolution 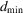, coefficients 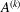, 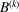, and 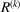 depend solely on the atomic type and can be pre-computed and tabulated. The number *K* of terms is defined by the required accuracy and the distance to which the approximation is built. We note that this allows the calculation of map values at any set of points, not only on a regular grid aligned with the coordinate axes.

In this work, we describe a computational algorithm for calculating analytical approximations to atomic Fourier images at any required accuracy, together with the gradients with respect to atomic model parameters needed for refinement. The aim of the algorithm is to determine the parameter sets in equation (1) that are as small as possible while providing the most accurate approximation. We also present its implementation in *CCTBX* (Grosse-Kunstleve *et al.*, 2002 ▸) and *Phenix* (Liebschner *et al.*, 2019 ▸), making the method accessible to the broader community and paving the way for its use in applications that require finite-resolution maps generated from atomic models. Using this algorithm, we provide tables of the coefficients for all atom types over a broad range of resolutions. The method is supported by numerical tests demonstrating that these approximations reproduce Fourier maps calculated by standard methods with high accuracy, while achieving runtimes comparable to those of the fast Fourier transform (FFT) approach or even faster.

## 2. Method

### 2.1. Images of atomic density

Originating from Vand *et al.* (1957 ▸), multi-Gaussian approximations to the atomic scattering functions are widely used, in their current form proposed by Agarwal (1978 ▸),
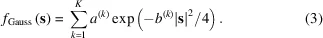
Here 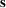 is a scattering vector, 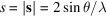 is its norm, and coefficients 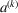 and 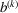 are specific to the chemical element and the experiment type. The larger value of the number *K* of the terms, the more accurate the approximation that can be achieved.

To model isotropic structural disorder, atomic densities of immobile atoms are convoluted with the Gaussian functions
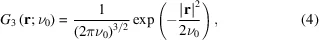
where 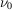 is related to the isotropic atomic displacement parameter *B* as 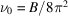. Owing to the convolution property of Gaussians
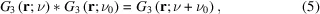
respective approximations to the atomic densities calculated with (3) get a form (Agarwal, 1978 ▸)
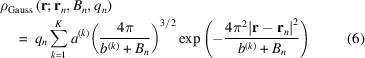
conventionally used to generate atomic density by their sums. Owing to the spherical symmetry of all functions considered here, in what follows we deal with their radial components, which we note by an overline, *e.g*.
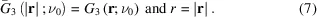
The radial component of the finite-resolution Fourier image of the density (6) for an atom with a unit occupancy and placed in the origin is calculated as
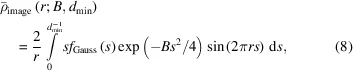
where 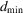 is the resolution cut-off, except for 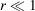 where it is calculated as
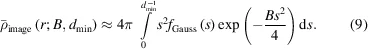
These images were used as the reference for obtaining 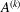, 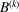 and 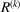 in (1).

Importantly, unlike the densities themselves, their finite-resolution oscillatory images cannot be approximated by a linear combination of centered Gaussian functions with positive or negative coefficients. The reason for this is that the Fourier transform of any such combination cannot exhibit the sharp cut-off at the required resolution limit that characterizes finite-resolution images.

### 2.2. Normalizing atomic images

**Figure 2:**
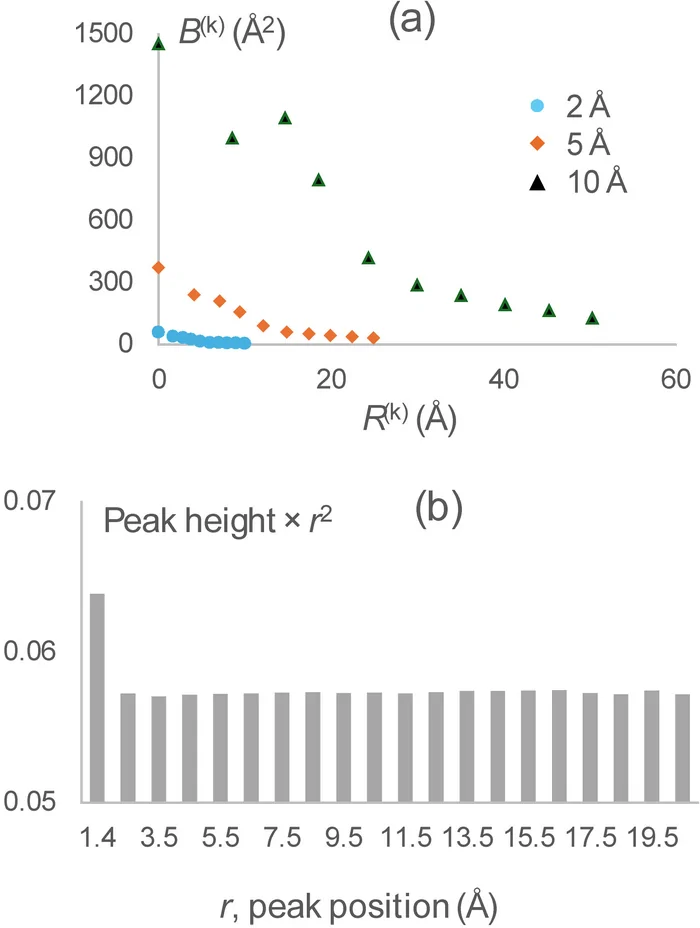
(*a*) Ten first coefficients 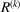 and 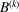 of the approximation to the images of a carbon atom at the resolutions 2, 5 and 10 Å. (*b*) Height of the ripples in the 1 Å resolution image of a carbon atom normalized by the value in its origin. The height is shown as a function of the distance to the origin, being multiplied by the square of this distance.

Images for different atoms at different resolutions look drastically different. Their magnitude is proportional to the number of electrons (for electron densities) and the positions of extrema of the radial components spaced apart approximately at 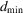 distance. Consequently, 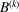 values grow as a square of a distance between these peaks. While at resolutions around 1 Å these coefficients are of the order of ten, at lower resolutions, 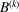 are two to three orders of magnitude larger [Fig. 2 ▸(*a*)]. Generally, numerical optimization favors variables being on the same scale, but may behave poorly when that is not the case.

**Figure 3:**
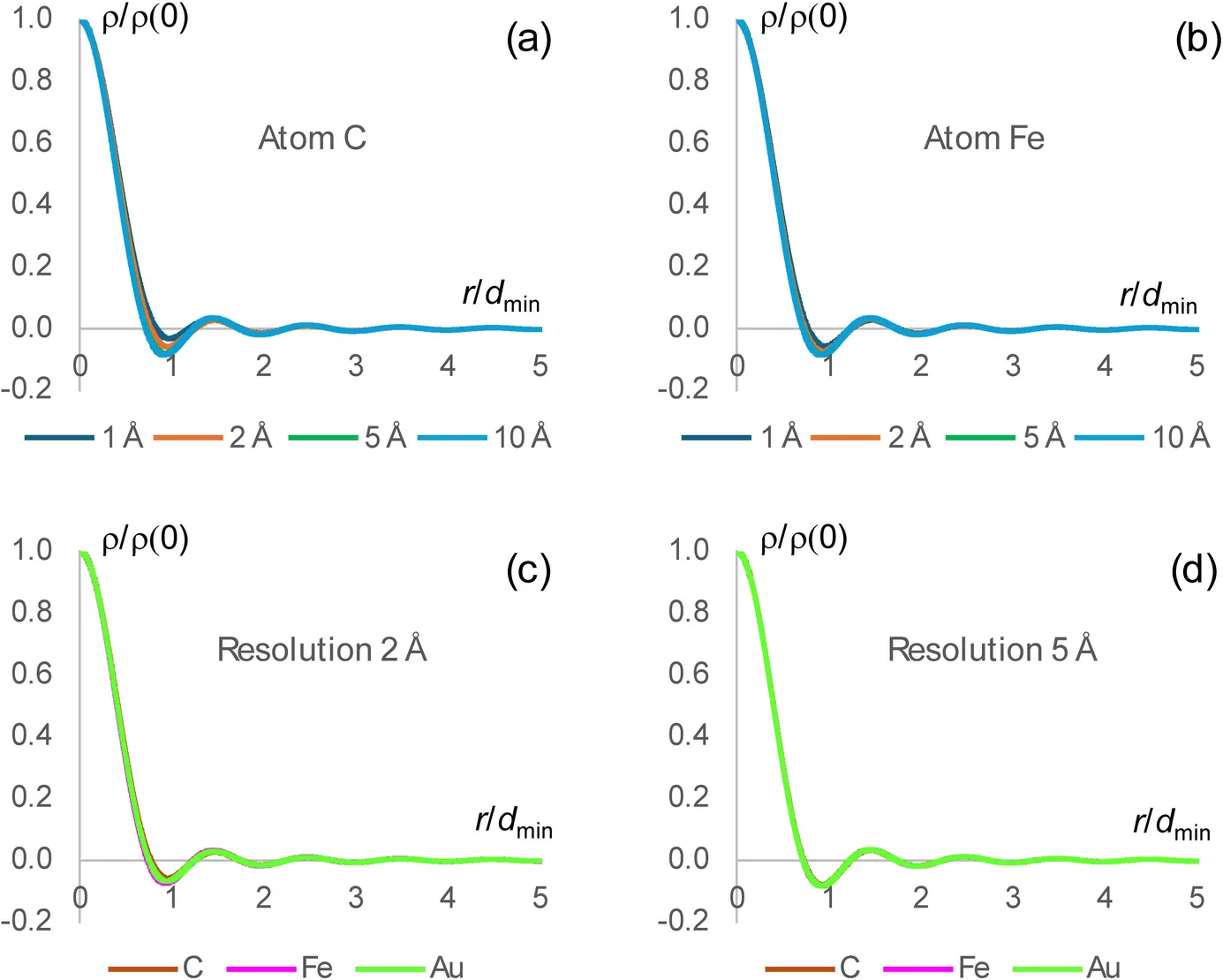
Atomic images scaled as in (10). Panels (*a*) and (*b*) show images for carbon (C) and iron (Fe) atoms at different resolutions. Panels (*c*) and (*d*) display images for carbon, iron and gold (Au) atoms at resolutions of 2 Å and 5 Å, respectively. Notably, despite the significant differences in both the types of atoms and the resolutions used in this example, all curves for the scaled images virtually coincide.

We observed that the finite-resolution images of immobile atoms expressed as a function of a normalized, dimensionless parameter 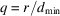 appear similar to that of *d*_min_ = 1 Å resolution. Moreover, an additional scaling of the curve magnitude to its value at the origin as
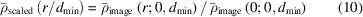
makes the normalized atomic images (10) similar to each other for all atomic types and for all resolutions (Fig. 3 ▸).

The utility of such image scaling is twofold. First, expectedly, it drastically improves the numerical procedures of the search for coefficients of the approximation (1) to the rescaled image (10). When the approximation coefficients 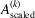, 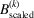 and 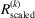 for a normalized curve 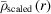 (10) at the interval 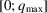 are determined, the coefficients for the original curve (1), that is on the absolute distance scale, in Å, and for the distance up to 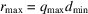, are obtained by reverting the scaling:
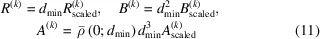
[see *e.g.*, equation (6) to understand the dependence of the coefficients 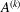 and 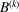 on the distance scale]. To keep the total integral of the function (‘atomic charge’), the 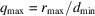 value has been recommended to be integer or half-integer (Urzhumtsev *et al.*, 2022 ▸).

Second, the similarity of the scaled images allows the reuse of their corresponding 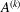, 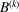 and 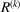 coefficients, once found for a given atom at given resolution, as a starting point for the search for coefficients for different atoms or/and resolutions, which greatly improves the results.

### 2.3. Approximation to finite-resolution atomic images

In this work, finite-resolution maps are calculated following (1) where 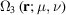 (2) appears instead of Gaussians in (6). In fact, Gaussian function (4) is a particular case of the function 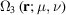 corresponding to 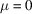. This function has its shape concentrated in a shell around a sphere of radius 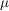 with a width defined by the parameter 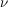. The key feature of these spherical shell functions is that, similar to (5), they do not change their shape after convolution with the Gaussian function (Urzhumtsev & Lunin, 2024 ▸),
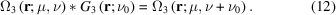
This is especially important for modeling atomic images with positional uncertainties, as this is done for atomic densities. Based on the properties of 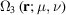, two approaches to calculating an approximation (1) to atomic images have been proposed (Urzhumtsev *et al.*, 2022 ▸).

In the first approach, for an atom *n* of a given type with 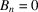 (an immobile atom) and a unit occupancy, one calculates its image 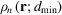 placed at the origin at the required resolution 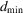, and then builds an approximation (1) to this image as
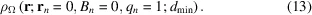
Approximation (1) to an atomic image at the same resolution with any 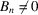 is then available using (1), (2) and (10). By construction, the constants 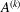, 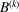 and 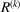 are the same for all atoms of the same chemical type.

In the second approach, one builds the 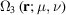 approximation (1) to the three-dimensional unit interference function
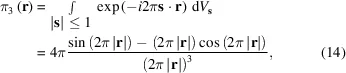
which is the image of a point scatterer at the resolution *d*_min_ = 1 Å (Urzhumtsev & Lunin, 2024 ▸). With (10) and other properties of 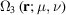 and of the interference function, this provides the coefficients of the approximation (1) to the image of any Gaussian function (4) at any chosen resolution 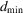 (Urzhumtsev & Lunin, 2024 ▸). Combining these images according to the *K*-Gaussians approximation (6) to the atomic densities, this gives the coefficients of an approximation (1) to the image of any atom with any isotropic 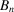 value at any resolution 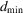 as a sum of the respective Gaussian images.

This latter approach, implemented in *ModQMap* program (Urzhumtsev & Lunin, 2025 ▸), is more general, because it computes a unique set of coefficients analytically adjustable to any resolution, but is less attractive computationally. The number of terms for an image of each individual Gaussian component is roughly the same as the number of coefficients for the whole atomic image in the former approach. Thus, the number of terms in (1) is about *K* times larger (usually about 5–6 times) compared with the number for the former approach; the same holds for the CPU time. Since CPU time is especially important for refinement where the calculation of maps and their derivatives is repeated many times, we programmed the former method of the approximation.

### 2.4. Approximation protocol

#### 2.4.1. Obtaining approximation parameters for a given image

The set of parameters 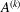, 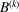 and 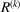 for an image of a given atom at the specified resolution 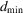 are obtained by fitting function (1) to the reference atomic image (8–9) as described by Urzhumtseva *et al.* (2023 ▸). This process is governed by two principal parameters: 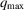, defining the distance limit 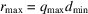 from the atom’s center up to which the approximation is calculated, and the maximum allowed approximation error, which is computed as the absolute value of the difference between the reference curve and its approximation calculated over the interval 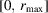. Their values and the coefficients 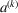 and 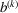 of the scattering function (3) are the input parameters to the procedure.

Calculation of optimal 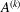, 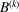 and 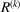 occurs in two steps: assignment of approximate values of the coefficients, according to the curve’s extrema position and shape, followed by their refinement to minimize the least-squares discrepancy between the reference atomic image (8–9) and its approximation (1). The scaled atomic image 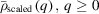, at a given resolution 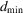 is analyzed in increasing order of the argument value from 0 up to the distance limit slightly beyond 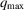 (see Section 2.4.3); then, for a given position of a local extremum, a new term is noted if is above the predefined maximal allowed deviation (see also Section 2.4.3). After the whole interval 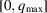 is analyzed, the curve calculated using (1) is subtracted from the reference curve. Optionally, more iterations can be done when the residual curve is scanned for local extrema again, and, if any found above 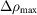, an additional term is added that optimally fits it.

Numeric minimization of the least-squares difference between the reference curve and its approximation (1) is done by the L-BFGS-B method (Liu & Nocedal, 1989 ▸), resulting in the coefficients 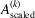, 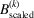 and 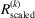 for the scaled image. As is typical for a local optimization, the accuracy of the approximation with these coefficients largely depends on their initial values. This creates room for several combinations of initial assignment of the coefficient values and their following refinement to find the best approximation. There are three algorithms used to initiate the procedure:

Algorithm 1: Parameters of all assigned terms are refined after the whole curve is analyzed.

Algorithm 2: Parameters of all terms are refined after the whole curve is analyzed, except the parameters of the first term approximating the central peak, which are refined as soon as they are estimated.

Algorithm 3: Parameters of each term are refined, together with the previously defined parameters, as soon as they are estimated.

The computation time in each of the three algorithms is dominated by that of refinement of the coefficient values, increasing with the number of the coefficients. For this reason, the third algorithm requires more CPU time but sometimes gives a more accurate approximation.

At the end of the procedure, the coefficients 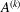, 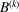 and 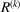 for the original, unscaled atomic image are calculated according to (11).

#### 2.4.2. Tabulating parameter values

For a given atom, coefficients of the approximation can either be calculated as needed for a specific resolution 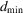 or pre-calculated and tabulated in advance for a set of atoms (*e.g.* the entire periodic table) and for a range of resolution values, allowing them to be reused later as necessary. The latter may be useful for highly repetitive calculations such as atomic model refinement, where on-the-fly calculation of image approximations may quickly become a computational bottleneck. For tabulating over the resolution range from 1 up to 2 Å, the step 0.005 Å has been chosen such that when moving between adjacent resolutions, the value of the atomic image in the origin changes by less than 1% of the peak value. Beyond 2 Å the step can be kept at 0.01 Å, which is the accuracy of the typically reported resolution values.

Calculating approximation coefficients for a series of images (*e.g*. when tabulating them) has an additional advantage due to the similarity of the scaled images (10), which can help in finding an approximation more accurately and faster compared with the search for the approximation for a single atom at a single resolution. Approximate values of 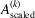, 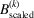 and 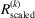 for a scaled image of a given atom at a given resolution may be taken as the initial values for refinement for the image of the same atom calculated at the adjacent resolution or as those for the image of another atom at the same resolution.

**Figure 4:**
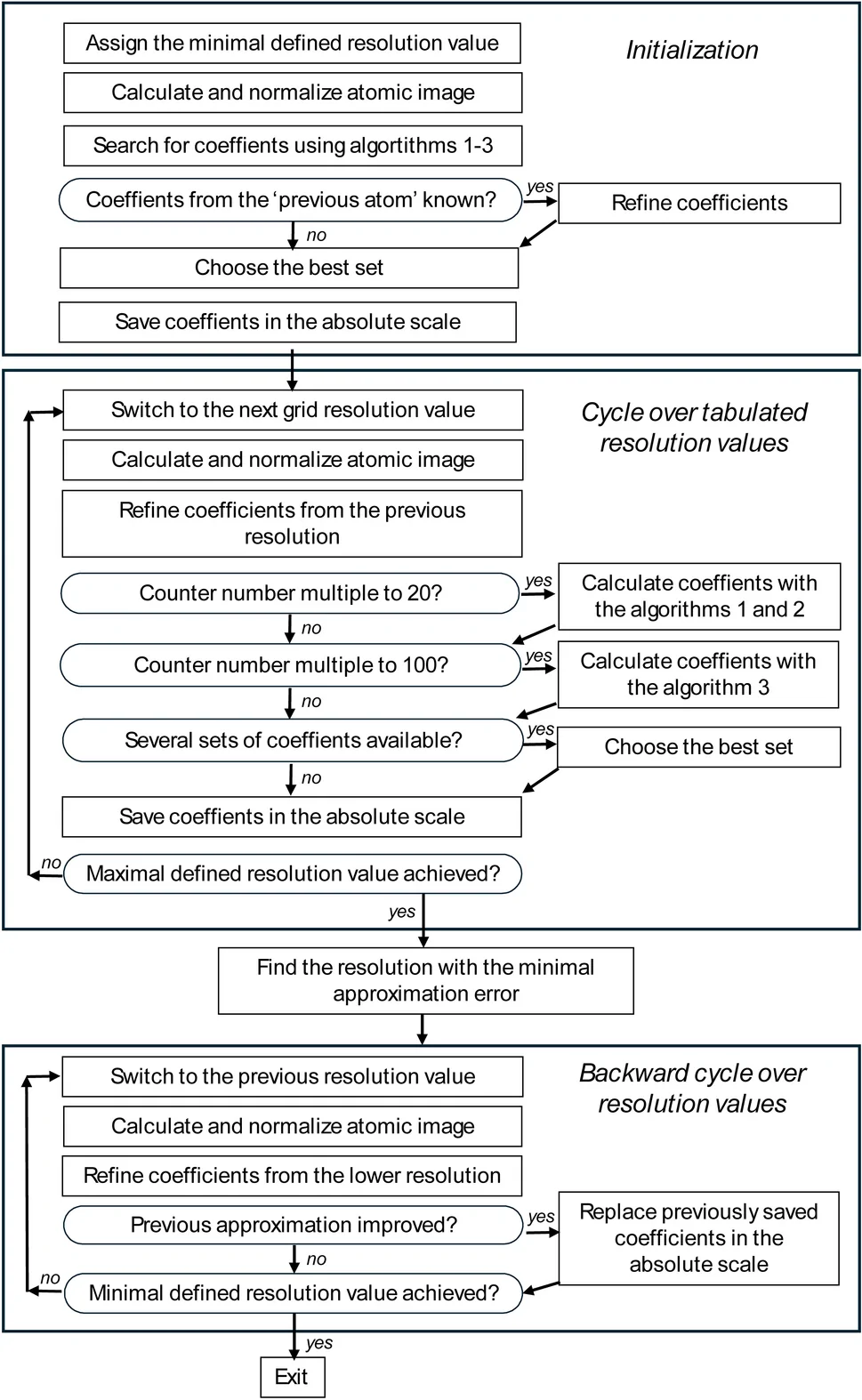
Flowchart of the procedure to determine the approximation coefficients (1) for a set of finite-resolution images of an atom of a given chemical type.

Fig. 4 ▸ outlines the procedure for finding optimal 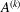, 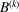, and 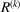 for a given atom at a series of resolutions. The procedure starts from the determination of the set of coefficients for the starting resolution value, as described in Section 2.4.1, assuming the resolution range is ordered by value from smallest to largest. Then it cycles over subsequent resolution values in the range. For each new resolution, the refined coefficients for the scaled image at a previous resolution are used as the starting values for refinement. This drastically accelerates refinement since these values are usually closer to the final values than the starting values defined by algorithms 1–3 (Section 2.4.1). At the same time, it is necessary to check whether such repeated recycling of initially defined coefficient values does not miss a better set of coefficients. For this, we additionally reuse the algorithms 1–3 (faster algorithms 1 and 2 are applied more frequently) and select the best result among all tested options.

In this protocol the coefficients for higher resolutions pass through a much smaller number of refinement iterations than those for lower resolution and therefore may be less accurate. To correct this, we scan over the whole set of resolutions for which the approximation coefficients have been calculated and identify the set of coefficients giving the minimal approximation error. Then, starting from this resolution and corresponding coefficients, the procedure is run in the opposite sense, taking the coefficients from the lower resolution and refining them to the higher resolution. If a better result is obtained, which is usually the case, the coefficients found during the forward resolution analysis are replaced by these new values.

As is done when moving from one resolution to another, a similar approach is applicable moving from one atomic type to another.

#### 2.4.3. Number of approximation terms

The number of terms in approximation (1) represents a trade-off between the runtime cost of the map calculation and the desired accuracy of the approximation. While more terms improve accuracy, fewer terms lead to faster calculations. The latter is especially important since atomic images decrease with distance much slower than the densities themselves [Fig. 2 ▸(*b*)], thus necessitating a larger distance cut-off value 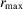 than is required to compute the exact density (Urzhumtsev *et al.*, 2022 ▸), and, consequently, a larger number of grid nodes for which the calculations should be done.

**Figure 5:**
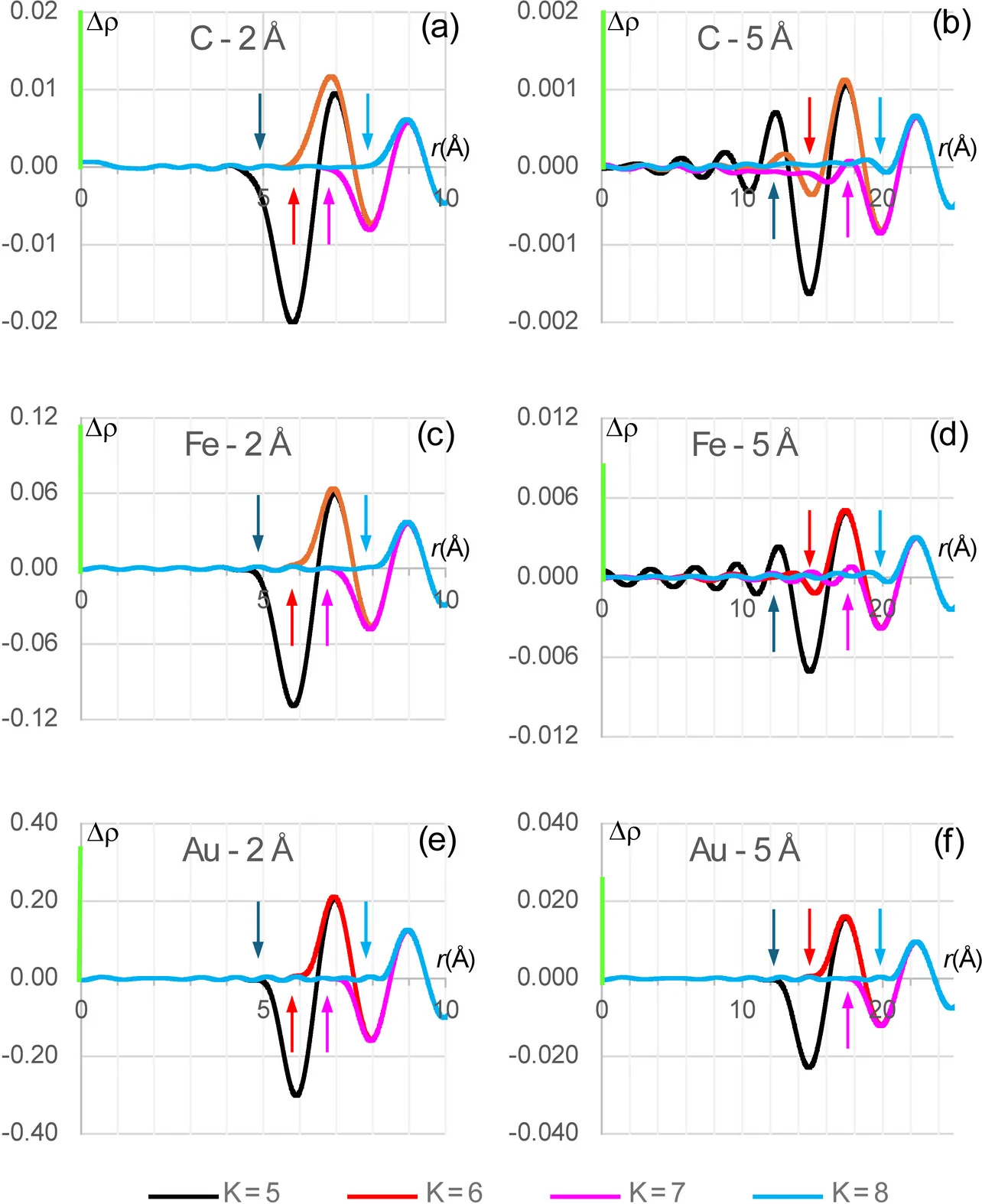
Difference, in e Å^−3^, as a function of the distance *r* to the atomic center, between the atomic image (8–9) for atoms of C, Fe and Au, and several approximations (1) to it, varying the number of terms *K*. A green bar at the axis shows the value 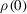 of the respective atomic image at its center divided by 100. 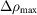 is computed for images of resolution 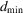 of 2 Å and 5 Å up to the distance limit 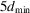. Colored arrows matching the curves’ colors indicate the value of the largest 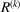 for the given approximation. Terms with 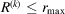 (considered here as 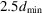, 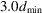, 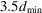 and 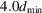) may be insufficient to produce an accurate approximation for the points close to 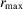 and one or two next terms beyond 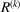 may notably reduce the error.

Intuitively, at least one term in (1) is required to represent the peak at the origin and at least one term is necessary to model each Fourier ripple, with two ripples per each distance interval of the length of approximately 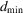. For such an approximation, the total number of terms with 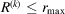
for an image at the resolution of 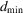 is about 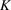 = 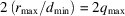. In fact, the atomic images (8) are calculated and analyzed up to the distance 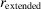 that is slightly larger than 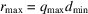. The extended approximation interval is used because it may happen that including one or a few terms with 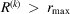 may notably reduce approximation errors at the end of the interval 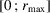. For example, for the image of a carbon atom at the resolution *d*_min_ = 2 Å, working with the approximations up to the distance limit *r*_max_ = 5 Å 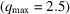, the two terms around this value are 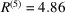 and 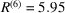, Table 1 ▸. The series cut at 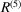 [Fig. 5 ▸(*a*), black curve] gives much larger errors at the upper boundary of the interval 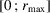 than a series completed by 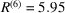 [Fig. 5 ▸(*a*), red curve]. Arrows of the respective color indicate these 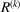 values.

After 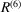 is included, this error is reduced at the interval [0; 5 Å] while it is still increased approximately at *r* approaching to 6 Å 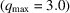. Similarly, to improve this approximation up to this limit, an extra term beyond 6 Å, 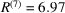, is required [Fig. 5 ▸(*a*), curve in magenta].

Depending on the atom type and resolution, a need for such extra terms may be weaker [Fig. 5 ▸(*c*, *e*, *f*)] or stronger [Fig. 5 ▸(*b*, *d*)]. For example, for the image of a carbon atom at the resolution *d*_min_ = 5 Å [Fig. 5 ▸(*b*)], compare the residual error up to the distances 12.5 Å 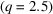 and 15.0 Å 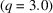 for the series with *K* = 5, 6 and 7 terms with 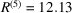, 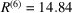 and 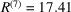, respectively.

The extended interval is defined as 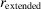 = 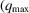 + 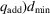 with an extra parameter 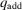 which, like 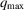, is an integer of a half-integer. Usually, adding a single term with 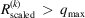 (or, respectively, 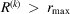) is sufficient. In most cases, this term belongs to the interval 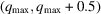. Occasionally a larger interval 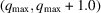 may be required, which also may allow identification of not one but two following terms, depending on the details of a particular curve.

To model all ripples in the scaled image up to the distance 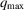 by (1), we define the maximal allowed starting approximation error to be equal to 90% of the absolute value of the last ripple at the scaled interval 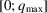. This guarantees that all local extrema of the curve are interpreted by the terms in (1). Refinement of the coefficient value usually reduces this starting approximation error to a value of the order of 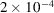 of the maximal value of the atomic image. When 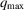 approaches 15, the starting accuracy defined as above falls below 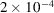 and cannot always be assured with a single term per ripple. However, a search for an approximation to atomic images and their analysis at such conditions is not of practical interest.

### 2.5. Variable-resolution map calculations for atomic models

Calculating a variable-resolution map for an atomic model requires assigning a resolution to each atom, which can be the same for all atoms or not. Next, for each atom type and its associated resolution, the approximation coefficients are retrieved from pre-calculated tables as described in Section 2.4.2. The map sampling procedure is then applied, consisting of a loop over all atoms to add each atom’s contribution to the map grid nodes that fall within a sphere of a given radius 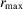 around the atomic center. The radius of this sphere depends on the resolution, the atomic displacement parameter and the purpose of the map being calculated. Choosing a specific radius value involves a trade-off between map accuracy and computation time. A larger radius increases the map’s accuracy; however, it also requires adding atomic contributions to more grid points, resulting in slower calculations. Possible choices of radii and their impact on map accuracy are discussed in Section 3. The local resolution, which needs to be defined for each atom, can be obtained using the *phenix.local_resolution* tool, which computes the resolution at each map voxel and can optionally transfer that information to each atom in the supplied atomic model using the extended mmCIF format field *_atom_site.phenix_resolution*, now supported by *CCTBX* and *Phenix*.

### 2.6. Gradient calculation for variable-resolution maps

One of the applications of variable-resolution maps is real-space refinement of atomic models, which aims to reduce the discrepancy between the experimental and model-calculated maps. As the simplest example, one may consider the least-squares target 
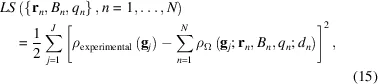
where the summation runs over grid nodes 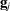 for which the maps are defined, and the local resolution values 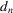 are considered as constants for which the coefficients of the approximation (1) have been calculated as described above. Efficient optimization methods require the calculation at least of the first derivatives of the function (2) with respect to all model parameters, *i.e.* the function gradient.

According to general theory (Baur & Strassen, 1983 ▸; Kim *et al.*, 1984 ▸; Lunin & Urzhumtsev, 1985 ▸), efficient algorithms should compute the gradient step by step, passing from one type of variable to the next in the opposite order to that used to calculate the score function. If *t*_f_ denotes the CPU time required to calculate a single value of the function itself, then, with such an algorithm, the CPU time needed to calculate all components of the gradient should not exceed 4*t*_f_, regardless of the number of parameters. In crystallographic reciprocal-space refinement, however, it was possible to achieve this even faster than *t*_f_ (Urzhumtsev *et al.*, 1989 ▸). For real-space refinement of atomic models, this requires derivatives of the variable-resolution-map (VRM) values with respect to the atomic model parameters, *i.e.* atomic coordinates, occupancies and *B* factors in the isotropic case. If *p_n_* denotes any of these parameters, the corresponding partial derivative with respect to *p_n_* is calculated as
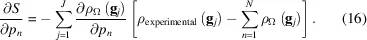
Here, for simplicity, we omit the list of parameters 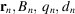. Obviously, each atom contributes only to a few grid nodes 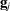 and therefore for each parameter 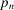
the outer sum in (16) includes only a small number of terms.

Because the atomic images 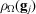 depend analytically on their parameters, all required partial derivatives can be expressed analytically. These derivatives are obtained in turn from the partial derivatives of 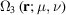 with respect to its own parameters. In what follows, 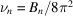, 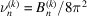, 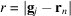 and, as previously, 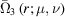 is the radial component of the function 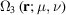. As a consequence,
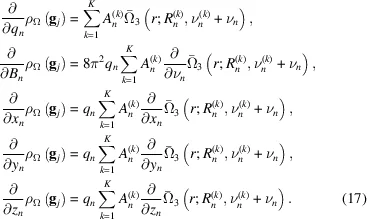
If 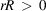, formulas (17) for the gradient use the expression for 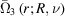 itself and
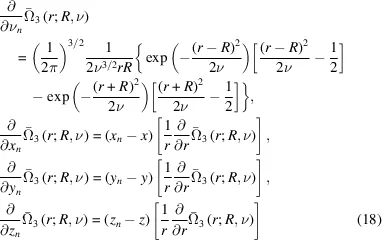
with
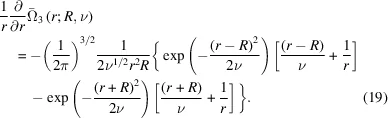
If 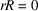, the function 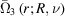 itself and its derivatives become
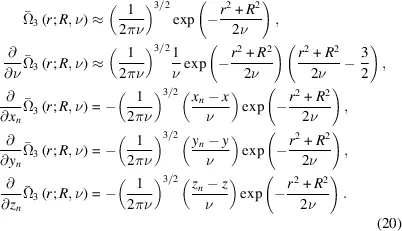
The module for the gradient (17) is a part of the software developed for the VRM calculation. Using it for real-space refinement of atomic models will be discussed separately.

## 3. Results

### 3.1. Tabulating image approximation parameters for X-ray and electron diffraction

As mentioned in Section 2.4.2, in highly repetitive calculations such as atomic model refinement, calculating image approximation parameters can become a computational bottleneck. Therefore, it is desirable to pre-calculate and store these values, allowing for easy and fast lookup rather than recomputing them on the fly. This also ensures better compatibility between different calculations, because these values are obtained by numerical fitting and may vary slightly depending on compilers and hardware. To this end, we calculated the 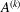, 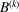 and 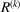 values for all entries (atoms and ions) in the X-ray (Waasmaier & Kirfel, 1995 ▸) and electron scattering function (Peng *et al.*, 1996 ▸; Peng, 1999 ▸) tables available in *CCTBX* across a resolution range from 1 to 10 Å, with the step size rationalized in Section 2.4.2.

These tables contain coefficients allowing the calculation of approximations up to the distance 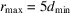. All the respective approximations contain one term per ripple (10 terms in total) and assure that the maximal difference between this approximation and the atomic image (8–9) is of the order of 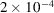 of the values of the atomic image in its center. An extended version of the tables up to the limit 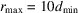 (22 terms per approximation) has been also calculated.

### 3.2. Test aims, data, metrics and expected sources of errors

In what follows, we describe numerical tests that aim to address two main questions pertinent to the practical use of the described methodology, namely: (1) the accuracy of the variable-resolution map (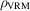) and (2) the time needed to compute it, both questions relative to the standard procedure of computing a finite-resolution map using fast Fourier transform (FFT) with structure factors calculated using the direct summation algorithm (referred to as reference, 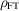). All maps were generated on a regular (128, 144, 160) grid. Integrals (8–9) were calculated numerically using the standard Simpson’s rule formula computed on a sufficiently fine regular grid on the inverse resolution (2000 equal intervals for 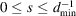).

For the tests with artificially constructed atomic models, including a two-atom model and a protein (PDB code 1crn), we placed these models into an orthogonal large unit cell of size 64 × 72 × 80 Å with *P*1 symmetry. Each of these models aimed at depicting particular source of errors detailed below. We used the 6-Gaussian approximations by Waasmaier & Kirfel (1995 ▸) for X-ray diffraction scattering functions, as available in *CCTBX*.

Three principal sources of discrepancies between 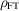 and 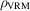 are

(1) errors in the map 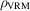 due to using approximation (1) as opposed to the similar map 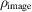 calculated with exact atomic images (8–9);

(2) including contributions within finite distances 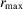 from atoms only as opposed to computing the atomic contribution to all grid nodes; and

(3) contributions arising from adjacent-by-periodicity cells; these contributions are inherently present in the 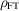 and crystallographic unit cells but are absent in atomic images computed in non-periodic volumes, 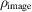, as in cryoEM.

To evaluate discrepancies between the reference and 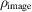 or 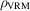 maps, we used the following metrics:

(*a*) mean and maximal values of the absolute error, defined as the absolute difference computed point by point between the maps for all selected grid nodes, denoted as e_abs, mean_ and e_abs, max_; and

(*b*) mean and maximal values of the relative error, defined as the absolute difference computed point by point between the maps for all selected grid nodes, divided by the value of 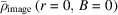 (8–9) of the heaviest atom in the model, denoted as e_rel, mean_ and e_rel, max_.

These errors were evaluated separately inside and outside the molecular region, defined as an accumulation of grid nodes within 2.5 Å radius from the atom positions.

### 3.3. Effect of the image truncation distance

#### 3.3.1. Two-atoms model

**Figure 6:**
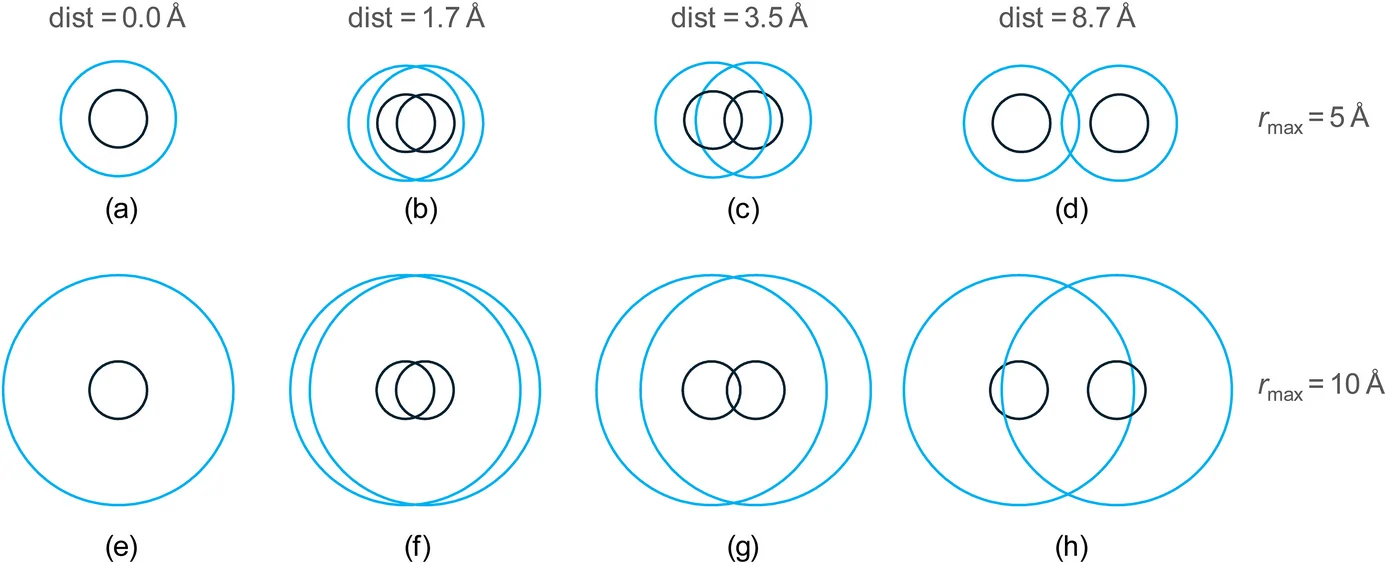
Schematic illustration of different atomic arrangements in some of the two-atom models. Black circles indicate atomic regions (masking radius *r*_atom_ = 2.5 Å). Blue circles with the radius 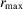 indicate regions that atoms contribute to.

In this test, we placed two carbon atoms with a zero *B* factor into the unit cell. The first atom was positioned at the center of the unit cell, while each of the three coordinates of the second atom was increased by *m*, where *m* = 0, 1, …, 5 Å. This resulted in six two-atom models, with the atoms spaced apart by 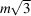
 Å and located exactly on the grid nodes. The molecular mask was calculated with *r*_atom_ = 2.5 Å. Three versions of 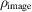 (8–9) were calculated with atomic contributions truncated at 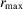 values of 3, 5 and 10 Å. Fig. 6 ▸ schematically illustrates some of these cases.

For the maps calculated with the exact images (8–9), when the two atoms coincide [Fig. 6 ▸(*a*, *e*)] the discrepancies between the two maps within the molecular region are attributed solely to contributions from neighboring cells, which the 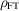 map includes but 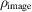 and 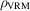 do not. These discrepancies, independent of 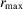, are relatively small, measuring 0.0011, 0.0033, 0.0004 for e_abs, mean_, e_abs, max_ and e_rel, max_, respectively. The error outside the molecular region is the sum of the former one and that of the image cut-off, with the latter one dominating. Obviously, for all cases with 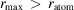, the error outside the molecular region decreases with 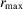 (Table 1 ▸, right part, column ‘0’).

When the distance between atomic centers is relatively small compared with 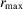 (*e.g.* the distance dist = 1.73 Å and 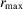 is equal to 5 and 10 Å), atomic images contribute to all points inside the molecular region [Fig. 6 ▸(*b*, *f*)] maintaining the same map difference (Table 1 ▸, columns ‘1’). For intermediate interatomic distance values, one atom can contribute or not to the maps for the second atom (Table 1 ▸, columns ‘2’, ‘3’, ‘4’) depending on the 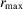 value [compare Fig. 6 ▸(*c*) with 6(*g*)]. For a large interatomic distance, for example 8.7 Å [Fig. 6 ▸(*d*, *h*)], the error inside the molecular mask increases (Table 1 ▸, columns ‘5’), but less for larger values 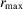 since the contributing atomic image 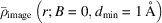 (8) is cut at a lower value. These numbers show that the contribution of atoms from neighboring cells can be ignored if the molecule is well isolated in the cell (placed in a sufficiently large box), as is usually the case for cryoEM studies.

Another important observation is that substitution of 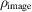 with 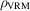 leads to a rather small difference with the maximal deviation of the order of 0.05% with respect to the value of the atomic image in its origin (data not shown). The main conclusion of these tests is that the principal difference between 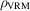 and 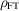 is due to the truncation of the atomic images.

#### 3.3.2. Parameters for VRM maps calculation

**Figure 7:**
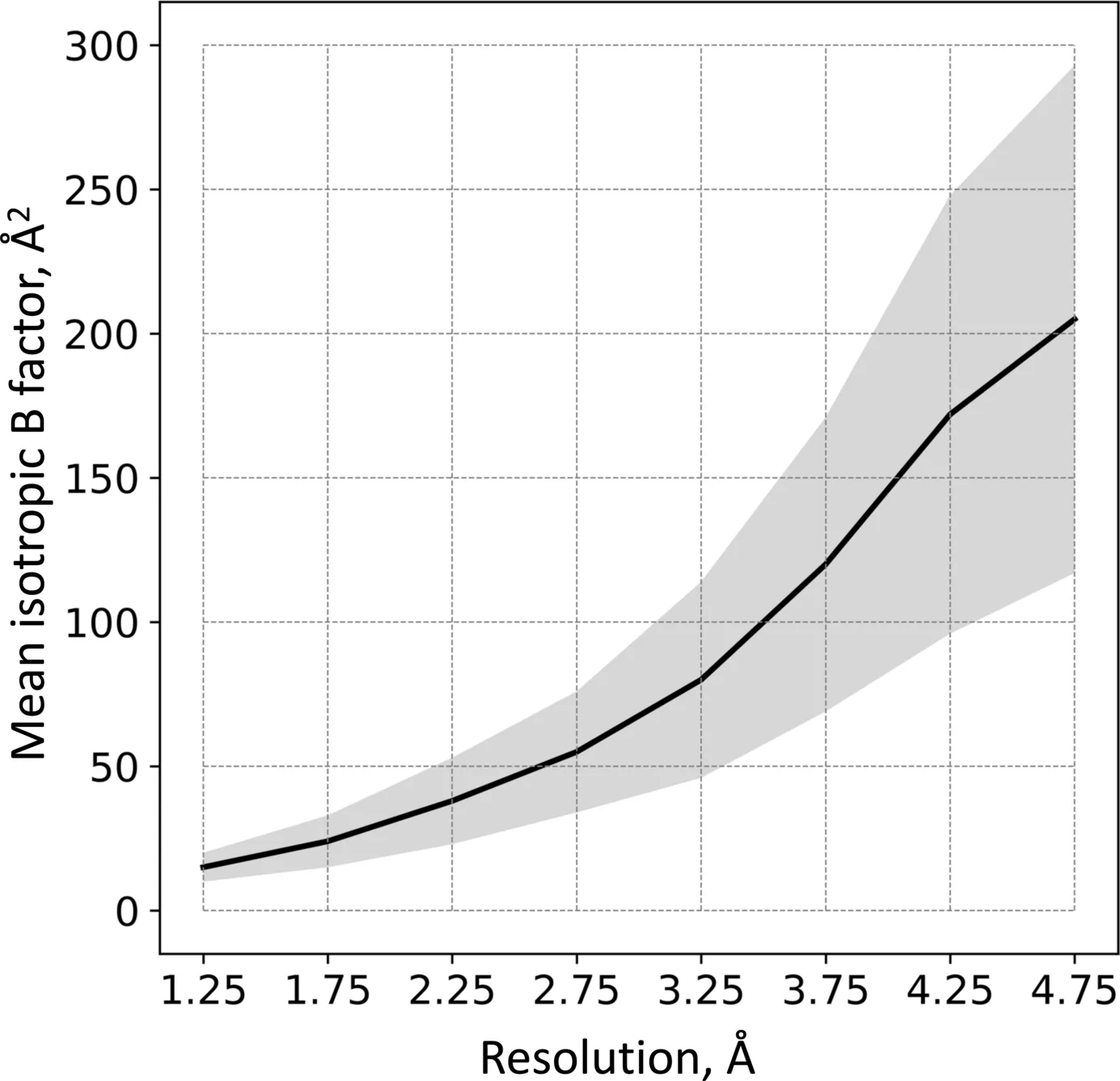
Structure-mean *B* factor as function of resolution for models in the PDB (black curve). The shaded area shows the standard deviation. Marks on the *x* axis show midpoints of resolution bins in which averaging of *B* factors was done: (1, 1.5), (1.5, 2),…, (4.5, 5) Å.

As pointed out in Section 2.4.3 and demonstrated in Section 3.3.1, the accuracy of 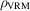 depends on the 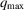 and 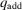 parameters, the choice of which, in turn, depends on the resolution and atomic *B* factors, as these two blur atomic images. Statistics over the structures in the Protein Data Bank (Berman *et al.*, 2000 ▸; Burley *et al.*, 2025 ▸) show the dependence of the structure-mean *B* factor on resolution, as depicted in Fig. 7 ▸. We used this as a guide to sample 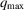 and 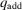 for pairs of (resolution 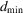, *B*) that follow this curve.

We observed that, as expected, using 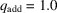 instead of 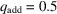 systematically improved the values of the statistical characteristics but rather marginally, and further increasing to 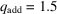 did not change them any further (data not shown).

**Figure 8:**
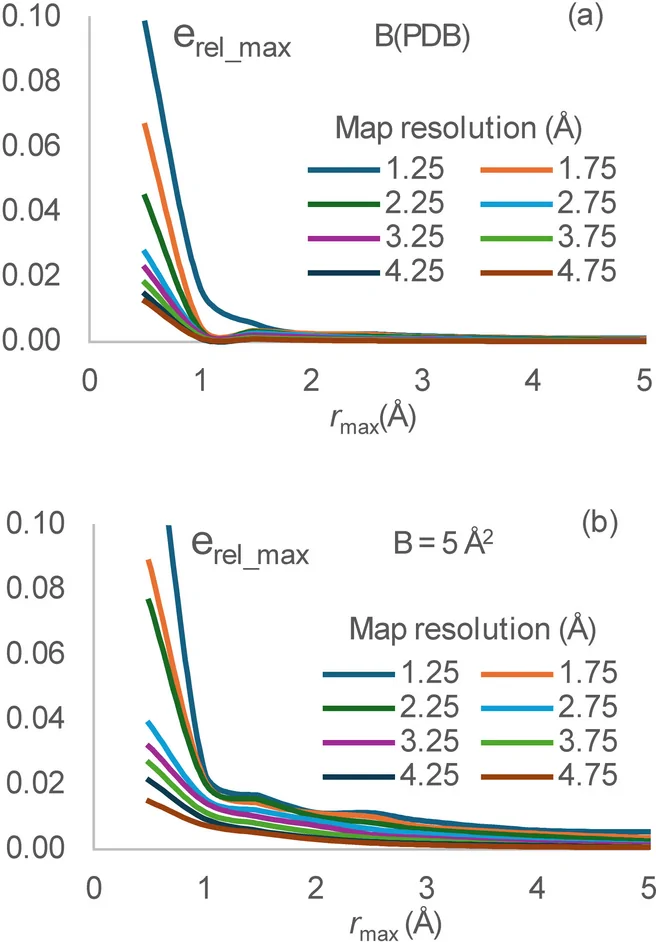
Discrepancy values between 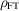 and 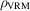 for the crambin model (PDB code: 1crn). (*a*) Relative error 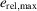 between 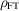 and 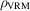 maps inside the molecular mask for the protein model, shown as a function of the image 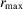; the maps are calculated for different map resolution 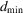 with 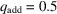; the *B* values are assigned according to main values from Fig. 7 ▸. (*b*) The same as (*a*) but with all *B* = 5 Å^2^.

We also observed that in all cases 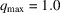 and 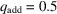 are sufficient to achieve cross-correlation CC_mask_ (Urzhumtsev *et al.*, 2014 ▸) between 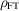 and 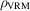 greater than 0.99 for a crambin model (Teeter, 1984 ▸; PDB code 1crn) placed in the center of unit cell box as defined in Section 3.2. There is no objective reason not to generalize this finding to any atomic model that follows Fig. 7 ▸. The maximum local map error is illustrated by Fig. 8 ▸(*a*).

Additionally, we performed calculations with 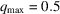 and 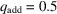. In this case, we go slightly beyond an approximation with a single Gaussian, 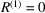, but the sum (1) includes also one term with 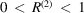. In all test cases, the masked cross-correlation CC_mask_ drops to 0.7–0.8, in the best case to 0.85, which is below our set goal of 0.99. This clearly demonstrates an advantage of the VRM approximation versus a simple Gaussian, and it indicates the need to include at least two or three terms with 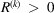, depending on the desired accuracy.

Using sharpened maps is common in cryoEM, in which case the mean refined *B* value may not follow the curve in Fig. 7 ▸, rather the refined *B* value may be well below 10 and typically close to zero; in this case 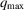 may need to be increased to 1.5–2.0 to maintain correlation match between 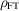 and 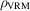 greater than 0.99; see Fig. 8 ▸(*b*) for the respective maximum local map error.

### 3.4. Runtime illustrations

The CPU time increases cubically with the distance 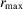, and inversely cubically with the map grid step. The choice of grid step depends on the specific task. A grid step smaller than 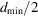 does not provide new information (Nyquist, 1928 ▸; Shannon, 1949 ▸).

In the following test, we compare the times needed to compute 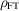 and 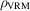 maps, computed on grids with a step of 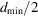 for a series of models with atom counts ranging from 500 to 50 000. The 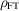 was computed using fast Fourier transform (FFT; Cooley & Tukey, 1965 ▸), with structure factors also calculated using FFT and all default parameters of *CCTBX*, at resolutions of 2 Å (most common for X-ray crystallography) and 4 Å (cryoEM), utilizing 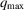 and 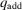 defined in Section 3.3.2. All models were placed in a *P*1 unit cell with dimensions such that the buffer between any atom of the model and the nearest adjacent cell is no less than 5 Å. The specific identity of these models is not of particular interest in the context of this test.

**Figure 9:**
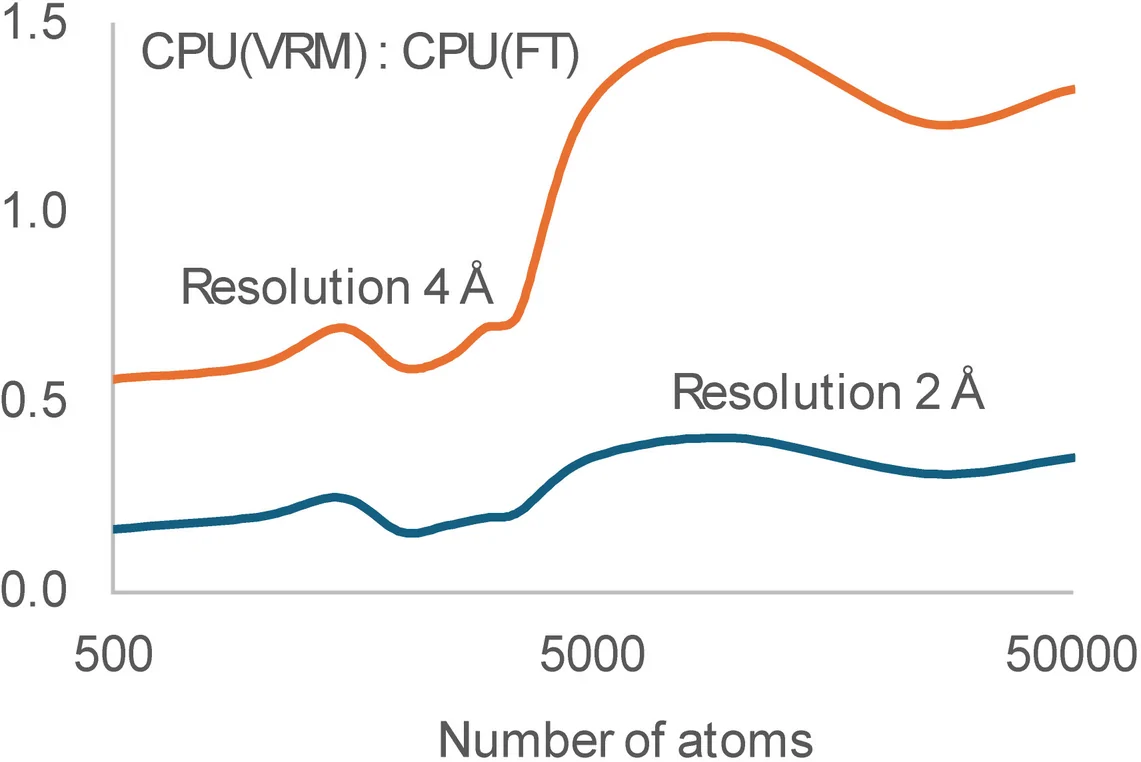
Ratio of the CPU time to calculate a map by VRM (this work) to that for a map calculation by the traditional three-step FT-based procedure. The ratio is shown as a function of the number of atoms for the maps calculated at a resolution of 2 and 4 Å with the default parameters as described in the text.

Fig. 9 ▸ shows the time ratios 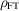: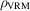 for these scenarios as a function of the number of atoms at two different resolutions. For a given resolution, the ratio increases slightly with the number of atoms (approximately logarithmically). The proposed method, applied at a reasonably high resolution of about 2 Å, not only accommodates variations in local resolution but also provides a gain in CPU time. Notably, there is a similarity between the curves calculated at different resolutions. However, at lower resolutions, there is no additional gain in CPU time (nor any significant loss).

We remind the reader that any gain in the calculation of the score function, essentially the model map here, translates into an equivalent gain in the calculation of its gradient (Lunin & Urzhumtsev, 1985 ▸).

### 3.5. VRM in action: local resolution estimation

Here, we return to the example shown in Fig. 1 ▸ of the *Introduction*[Sec sec1] and analyze its different aspects. The overall reported resolution of the map is 1.09 Å, whereas the local resolution varies across the map. When computing a model map for comparison with the experimental map, for example to assess model-to-map agreement using the cross-correlation coefficient CC_mask_ (Afonine *et al.*, 2018*a* ▸), which is a map-space analog of the crystallographic *R* factor (Booth, 1945 ▸), or to evaluate a refinement target based on matching the experimental and model maps, the question arises as to what resolution should be assigned to atom Fe 201 in chain A when computing its contribution to the simulated model map. In principle, this should be determined by the local resolution of the experimental map at that position.

**Figure 10:**
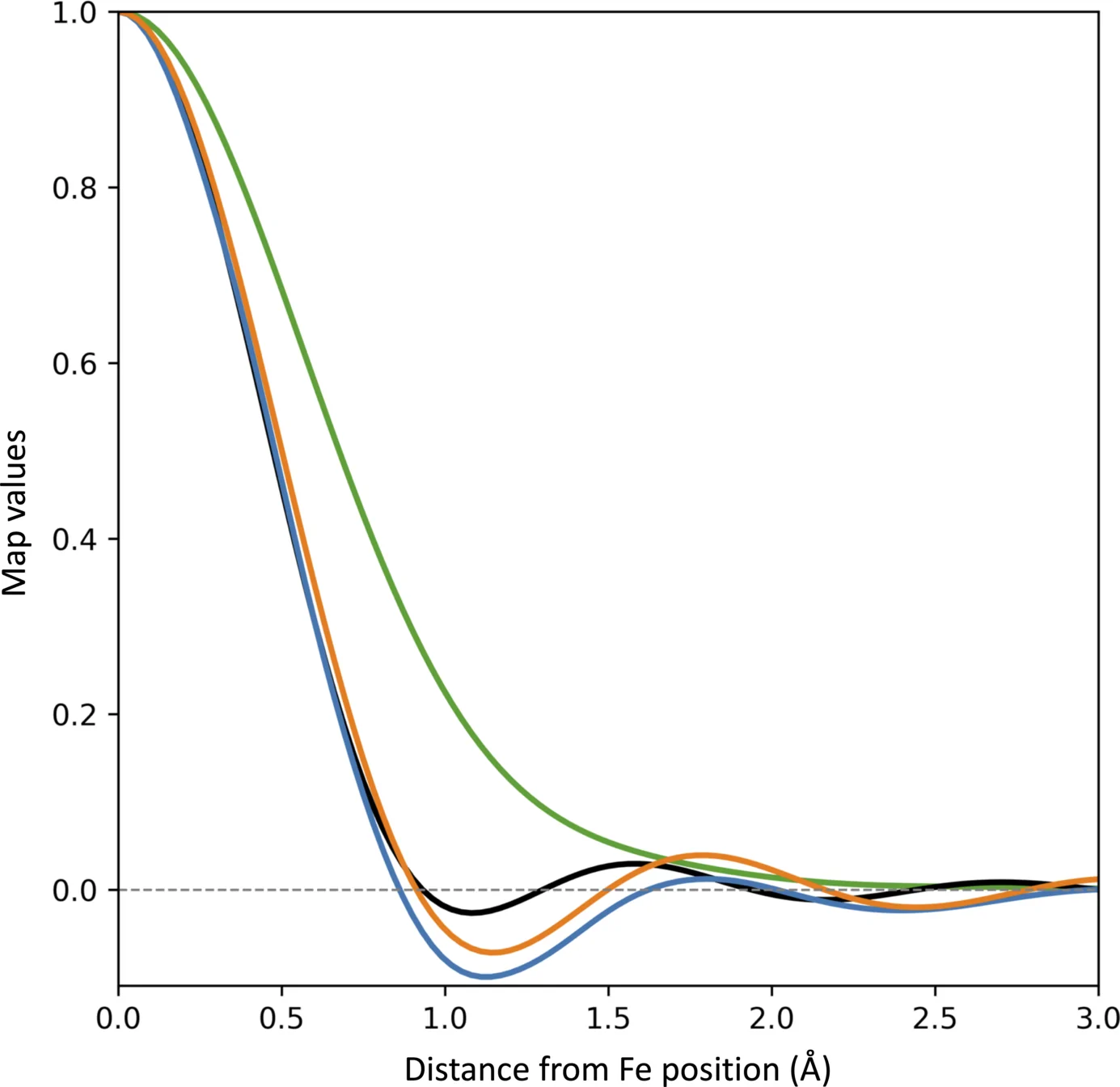
CryoEM data considered in Fig. 1 ▸, and still focusing on Fe 201 in chain A. Blue: spherically averaged cryoEM map profile originating from iron (0.0 Å) out to 3 Å. Orange: Fourier image of isolated Fe at 1.25 Å resolution with *B* = −6 Å^2^. Green: Fourier image of isolated Fe at 1.09 Å resolution with *B* = 17.22 Å^2^. Black: Fourier image of isolated Fe at 1.09 Å resolution with *B* = 0 Å^2^. All maps normalized by their values at the origin (maximum value).

To address this question, we computed the spherically averaged radial distribution of experimental map values centered at the iron position; this is shown as the blue curve in Fig. 10 ▸ (the same profile as in Fig. 1 ▸). In this case, it is reasonable to assume that the iron profile is not significantly affected by neighboring atoms, because the nearest atoms are more than 3 Å away, the resolution is better than atomic and the *B* factors are small.

Next, we calculated the Fourier image of Fe using equation (1), assuming the reported resolution of 1.09 Å and the *B* factor of 17.22 Å^2^ taken from the model file. The corresponding radial profile is shown as the green curve in Fig. 10 ▸. Clearly, the experimental (blue) and calculated (green) curves do not match well. Judging from their shapes, one possible explanation is that the *B* factor is suboptimally refined; indeed, the green curve appears noticeably more smeared.

This hypothesis can be tested by systematically varying the *B* factor and selecting the value that yields the best match between the green and blue curves, as measured by the *R* factor between the curve values. The corresponding optimized profile is shown as the black curve in Fig. 10 ▸ and is obtained with *B* = 0 Å^2^. Optimization of the Fe *B* factor substantially reduces the mismatch between the experimental profile and the atomic image calculated at 1.09 Å resolution. However, the agreement is still off, suggesting that the actual local resolution at this position differs from the nominal value of 1.09 Å.

We therefore performed a two-dimensional search over resolution and *B*-factor values, this time optimizing the agreement between the positions of the extrema of the experimental and calculated radial profiles, *i.e.* the positions of the peaks and dips. This yielded the orange curve in Fig. 10 ▸, corresponding to a resolution of 1.25 Å and a *B* factor of −6 Å^2^. The remaining small discrepancy between the blue and orange curves can be attributed to experimental errors, environmental effects and the anisotropic nature of atomic vibrations. The physically unrealistic *B*-factor value indicates possible sharpening being applied to the map. Interestingly, the resolution interpolated at the position of the Fe from the local resolution map, the result of *phenix.local_resolution* (Terwilliger; https://phenix-online.org/), is 1.23 A^2^, which is very close to the value obtained above.

This example illustrates how the VRM approach can be used to account for local resolution, and highlights the importance of accurate local-resolution estimates for achieving an adequate match between model-calculated and experimental maps, to identify physically meaningless *B* values in manipulated maps or hopefully to identify the correct values in the native maps.

### 3.6. VRM in action: map comparison

One of the applications of VRM maps mentioned in the *Introduction*[Sec sec1] is the validation of atomic models against cryoEM maps. This can be performed by comparing the experimental map to a map computed from an atomic model. CC_mask_ is one of the metrics that can be used to quantify the discrepancy between the two maps. Because CC_mask_ uses all atomic model parameters, *Phenix* employs it as a default choice. A major drawback of this metric is that its calculation assumes uniform resolution across the entire map, in line with the three-step procedure used to compute model-based maps as described in the *Introduction*[Sec sec1]. VRM maps remove this limitation, because the resolution of a VRM map can vary at the atomic level.

To illustrate the effect of including the local resolution into CC_mask_ calculation, we selected an atomic model of the actin-bound myosin and its corresponding cryoEM maps (PDB code: 6c1d; EMDB code: 7329). Because both resolution and atomic displacement parameters (ADPs) act as smearing factors in map calculation, they can be correlated; thus, refined ADPs may partially compensate for the lack of accounting for local resolution. Therefore, a rigorous demonstration of the impact of including local resolution in CC_mask_ would require refinement of the atomic model using model-calculated maps computed with and without accounting for local resolution. The former is not currently available in standard model-refinement software and is the subject of future work by the authors. We therefore resort to the following numerical experiment.

**Figure 11:**
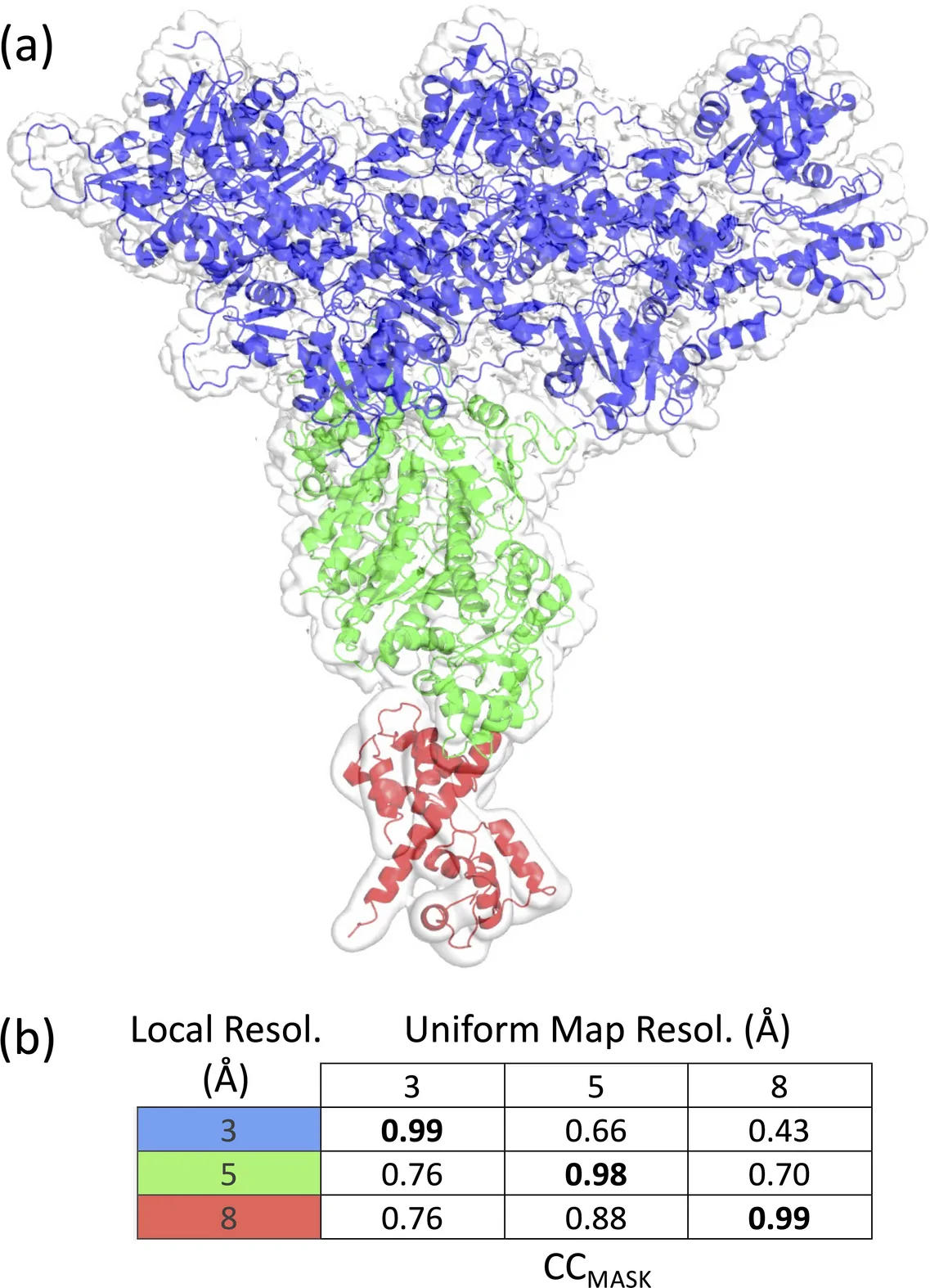
(*a*) Actin-bound myosin model in a VRM map computed using the local resolution assigned per atom for three regions: 3 Å (blue), 5 Å (green) and 8 Å (red) (all atoms within each region share the same resolution of 3, 5 and 8 Å, respectively). (*b*) CC_mask_ correlation for combinations of local resolution in the control map and the uniform resolution used for the model-calculated map. Note the remarkably low value of CC_mask_ = 0.66 for the region of the control map with 3 Å local resolution when the uniform resolution was assumed to be 5 Å. Values in bold on the diagonal indicate overlap of local and uniform resolutions and are expected to be nearly 1.0 by the design of the test.

First, we note that for this structure the local resolution clusters well into three spatial regions of approximately 3, 5 and 8 Å, as shown in Fig. S5 of Mentes *et al.* (2018 ▸). To mimic this, we assigned a resolution of 3 Å to atoms in chains A, B, C, D and E; a resolution of 5 Å to atoms in chain P residues 6–700; and a resolution of 8 Å to atoms in chain P residues 701–734 and to the entire chain R [Fig. 11 ▸(*a*)]. We then computed a VRM map using these assigned per-atom resolutions together with the deposited atomic model; we refer to this map as the control map (simulated ‘experimental map’). Next, we performed three CC_mask_ calculations in which we computed the model map using a uniform resolution of 3, 5 and 8 Å, respectively. In this numerical experiment, the only discrepancy between the control map and the model-calculated map is the local resolution accounted for in the control map and not accounted for in the model-calculated map. Consequently, deviations of CC_mask_ from the ideal value of 1.0 arise solely from the lack of considering the local resolution in the model-simulated maps. The purpose of this experiment is therefore to illustrate how CC_mask_ can produce inaccurate agreement scores when local resolution varies across the experimental map and is not included in the model map calculation.

Fig. 11 ▸(*b*) shows that nearly perfect correlation (around 1.0) is observed in regions where the control map resolution matches the corresponding uniform resolution used to compute the model map, as expected from the construction of the experiment. In the worst-case scenario, where the mismatch between the control map local resolution and the uniform resolution is maximal, the CC_mask_ correlation can be as low as approximately 0.4–0.6. Overall (computed using the whole model) CC_mask_ values are 0.95, 0.70 and 0.47 for resolutions of 3, 5 and 8 Å, respectively.

## 4. Discussion

In this manuscript, we describe the implementation of methods in *CCTBX* and *Phenix* that enable the calculation of finite-resolution maps, where the resolution can vary from atom to atom within the molecular volume.

Numerous self-consistent tests presented here demonstrate that VRM maps accurately match those traditionally computed using FFT, which served as the reference when the resolution is distributed uniformly. When analyzing approximations to the finite-resolution atomic images, we observed a similarity of images of all atoms at all resolutions after an appropriate scaling, which is interesting as an independent result by itself. Using this similarity allowed us to develop a practical algorithm to construct accurate approximations, with a single term per ripple.

Using the implementation of this algorithm in *CCTBX*, we have calculated and tabulated the coefficients of such approximations for all elements with known X-ray and electron scattering factors and for all resolutions in the range from 1 to 10 Å with a fine irregular grid step, smaller at higher resolutions. This allows for faster and non-redundant calculations using *Phenix* or other software. Given the approximation algorithm available on its own, new approximations can be routinely constructed for other conditions, for example at subatomic resolutions, and for other scattering functions.

Finally, this work led to the addition of resolution as an atomic property in mmCIF files (specifically, the *_atom_site.phenix_resolution* field) generated by *Phenix*. This simplifies the dissemination and exchange of this information between *Phenix* programs where it is needed. This parameter describes the map used to construct the model, not the structure itself.

This implementation also includes analytic expressions for the gradients of model-calculated maps with respect to atomic model parameters, enabling their use in atomic model refinement.

All code and Python scripts used to generate the test results are available in the *maptbx* module of the *CCTBX* library.

## Figures and Tables

**Table 1:** Discrepancy measures between 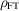 and 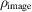 maps for six two-atom models Statistics are shown separately for the points inside and outside the molecular region. Error metrics are defined in Section 3.1. Shift defines the distance between the two atoms by the respective number of ångströms following each of the three axes (see Section 3.3.1).

	Inside						Outside					
Shift	0	1	2	3	4	5	0	1	2	3	4	5
*r*_max_ = 3 Å												
e_abs,mean_	0.0011	0.0105	0.0160	0.0127	0.0072	0.0045	0.0011	0.0007	0.0008	0.0008	0.0008	0.0008
e_abs,max_	0.0033	0.0565	0.0482	0.0436	0.0217	0.0116	0.1122	0.0791	0.0967	0.0855	0.0858	0.0670
e_rel,max_	0.0004	0.0060	0.0052	0.0047	0.0023	0.0012	0.0120	0.0085	0.0103	0.0091	0.0092	0.0072

*r*_max_ = 5 Å												
e_abs,mean_	0.0011	0.0010	0.0027	0.0067	0.0069	0.0045	0.0011	0.0007	0.0008	0.0008	0.0008	0.0008
e_abs,max_	0.0033	0.0027	0.0219	0.0220	0.0217	0.0116	0.0433	0.0425	0.0358	0.0428	0.0399	0.0426
e_rel,max_	0.0004	0.0003	0.0023	0.0024	0.0023	0.0012	0.0046	0.0045	0.0038	0.0046	0.0043	0.0046

*r*_max_ = 10 Å												
e_abs,mean_	0.0011	0.0010	0.0010	0.0009	0.0007	0.0010	0.0010	0.0006	0.0007	0.0007	0.0007	0.0007
e_abs,max_	0.0033	0.0027	0.0030	0.0028	0.0024	0.0063	0.0120	0.0109	0.0104	0.0104	0.0102	0.0109
e_rel,max_	0.0004	0.0003	0.0003	0.0003	0.0003	0.0007	0.0013	0.0012	0.0011	0.0011	0.0011	0.0012
